# Complete Sequence and Analysis of Coconut Palm (*Cocos nucifera*) Mitochondrial Genome

**DOI:** 10.1371/journal.pone.0163990

**Published:** 2016-10-13

**Authors:** Hasan Awad Aljohi, Wanfei Liu, Qiang Lin, Yuhui Zhao, Jingyao Zeng, Ali Alamer, Ibrahim O. Alanazi, Abdullah O. Alawad, Abdullah M. Al-Sadi, Songnian Hu, Jun Yu

**Affiliations:** 1 Joint Center for Genomics Research (JCGR), King Abdulaziz City for Science and Technology and Chinese Academy of Sciences, Riyadh, Saudi Arabia; 2 CAS Key Laboratory of Genome Sciences and Information, Beijing Institute of Genomics, Chinese Academy of Sciences, Beijing, China; 3 National Center for Genomics Research (NCGR), King Abdulaziz City for Science and Technology, Riyadh, Saudi Arabia; 4 Department of Crop Sciences, Sultan Qaboos University, AlKhoud, Oman; Universidad Miguel Hernández de Elche, SPAIN

## Abstract

Coconut (*Cocos nucifera* L.), a member of the palm family (Arecaceae), is one of the most economically important crops in tropics, serving as an important source of food, drink, fuel, medicine, and construction material. Here we report an assembly of the coconut (*C*. *nucifera*, Oman local Tall cultivar) mitochondrial (mt) genome based on next-generation sequencing data. This genome, 678,653bp in length and 45.5% in GC content, encodes 72 proteins, 9 pseudogenes, 23 tRNAs, and 3 ribosomal RNAs. Within the assembly, we find that the chloroplast (cp) derived regions account for 5.07% of the total assembly length, including 13 proteins, 2 pseudogenes, and 11 tRNAs. The mt genome has a relatively large fraction of repeat content (17.26%), including both forward (tandem) and inverted (palindromic) repeats. Sequence variation analysis shows that the Ti/Tv ratio of the mt genome is lower as compared to that of the nuclear genome and neutral expectation. By combining public RNA-Seq data for coconut, we identify 734 RNA editing sites supported by at least two datasets. In summary, our data provides the second complete mt genome sequence in the family Arecaceae, essential for further investigations on mitochondrial biology of seed plants.

## Introduction

The plant mitochondrial (mt) genome is considered as a remnant of an ancestral α-proteobacterium that was symbiont in its eukaryotic common ancestor [1]. It is involved in cellular energy production by respiration and various cellular function regulations, such as homeostasis, apoptosis, and metabolite biosynthesis [2]. Since the first mt genome of land plants was published (*Marchantia polymorpha*, liverwort) [3], there had been 303 mt genomes available until December 9, 2015 in the NCBI organelle database [4]. Plant mt genomes have several characteristics that make them important for evolutionary studies. First, plant mt gene contents are highly variable across plant taxa [5], obtaining genes from both plastid and nuclear genomes from intracellular transfer [6–8], as well as other species via horizontal transfer [9, 10]; plant mt genes can also be transferred to their nuclear genomes [11]. Second, plant mt genomes evolve more rapidly in their structure, but slower in their primary sequence [12, 13] as compared to both the chloroplast (cp) and nuclear counterparts. The genome-size expansion of plant mt genomes primarily reflects the increase of intronic and intergenic DNA [13] as plant mt genomes have dramatically lower mutation rates when compared to both their cp and nuclear counterparts [14, 15]. Third, plant mt genomes have a large number of copies per cell and show a remarkable amount of rearrangements [16]. A recent study has also shown that copies of the *Silene noctiflora* mt genome can be gained or lost, and the fact emphasizes evolutionary difference among them, the mt, the cp, and the nuclear genomes [17]. Fourth, plant mt genomes have a large number of intron-containing genes; some of them need trans-splicing to produce complete transcripts [18]. Fifth, plant mt genomes have a high frequency of RNA editing that contributes to functional conservation for the mt proteins [19, 20]. In plants, RNA editing affects mitochondrial and plastid transcripts by site-specific modification of cytidines-to-uridines and the reverse [20–23]. Taken together, the characteristics of plant mt genomes highlight difficulty of sequence assembly and analysis. Recently, we have released a mt genome assembly of date palm (*Phoenix dactylifera*) as the first one of the palm family [24], and now we add another, that of the coconut (*Cocos nucifera* L.), as the second of the palm family.

*C*. *nucifera* or coconut is one of the most economically important crops in tropics, serving as a source of food, drink, fuel, medicine, and construction material [25]. Although the plant has significant economic values as a significant crop, there have been a limited number of studies on its genome. Based on a flow cytometric analysis, a diploid genome of coconut is 5.966 ± 0.111 pg or 5.757 Gb in size, i.e., its haploid counterpart is 2.8785 Gb [26]. Genome sequencing data also supports this estimate, showing a genome size of ~2.6 Gb with 50% to 70% repeat contents [27]. Recently, several coconut transcriptomic studies have been reported [28–30], providing datasets for *de novo* transcriptomic assembly and other molecular studies. The coconut cultivars are generally classified into two types, the Tall and the Dwarf. In this study, we present the result for the first coconut mt genome of the Oman local Tall variety. We first acquired high-throughput sequences of total cellular DNA using the Roche/454 platform and assembled them into a complete chromosome, and then corrected some of the sequence variations using Illumina HiSeq data. We also analyzed the genome assembly using transcriptomic data for genome structure and functional genes based on various comparative genome analysis tools.

## Materials and Methods

### Plant materials

Fresh green leaves from an adult coconut plant of the Tall cultivar located at Salalah, Dhofar Governorate, Oman, were collected, washed with double-distilled water, and frozen immediately in a liquid nitrogen container. The farm is owned by one of the co-authors of this work, Dr. Abdullah M. Al-Sadi, who is employed by Sultan Qaboos University and to whom future inquiry should be addressed. This study does not involve endangered or protected species and does not require specific permission from regulatory authority concerning wildlife protection. After being transported to the laboratory, these samples were stored in -80°C freezers until use.

### Genomic DNA isolation and sequencing

The raw coconut mt genome sequences were extracted from those produced as part of the Palm Plant Genome Project (a joint effort between KACST and BIG, CAS). Genomic DNA was isolated from 50g fresh leaves according to a CTAB-based method [31]. 5mg purified DNA was used for library construction for both single-read and paired-read libraries with 3kb and 8kb insert sizes according to the manufacturer’s manual for GS FLX Titanium. The libraries were amplified and sequenced on the Roche/454 GS FLX platform. All Roche/454 data was deposited at BIGD database (http://gsa.big.ac.cn, CRX007340 and CRX007339). The same purified DNA sample was also used for constructing the Illumina HiSeq libraries. HiSeq paired-end (< = 500bp) and mate-pair libraries (1kb to 8kb) were constructed using the Illumina Simple Paired-End Library and Mate-Pair Library Preparation Protocol, respectively. The libraries were sequenced by Illumina HiSeq 2000 platform. HiSeq data used for coconut mt genome correction was deposited at BIGD database (CRX007360, CRX007361 and CRX007362).

### Sequence assembly and validation

We first assembled total reads from 13 single-read datasets and 12 paired-read datasets into 573,893 contigs using Newbler 2.6 (with “-a 0” option and default for others), a *de novo* sequence assembly software. We then aligned the assembled contigs to 234 published land plant mt genomes downloaded from NCBI organelle database at September 16, 2015 using BLAST (identity> = 80%, E-value< = 10^−5^ and overlap percent> = 30%) [32–34]. We next used 353 annotated contigs (length ranging from 102bp to 49,695bp with median size in 399bp) to build scaffolds using bb.454contignet and manually checked based on contig coverage and spanning reads in Newbler assemblies [35]. We finally obtained a single scaffold of 678,112bp in length without gaps from 143 overlapping contigs.

To correct the sequence errors that are unique to the Roche/454 platform in the assembly, such as homopolymers (characteristic of the pyrosequencing), we used HiSeq paired-end data (180bp insert size) and bowtie2 (version 2.2.4) [36]. The consensus sequence was obtained by using samtools (version 1.2) [37, 38] and bcftools (version 1.2) [39]. The length became 678,133bp after this correction. As a byproduct, we identified several pseudogenes due to frame-shifts caused by homopolymers. We checked the final assembly manually based on Roche/454 and HiSeq paired-end data using IGV software (version 2.3.61) and revised 687 loci with 528 indels and 159 SNPs [40, 41]. Finally, we obtained a new length of 678,653bp with average sequence depths of ~42x for Roche/454 data and ~1788x for HiSeq data. We checked this assembly using HiSeq mate-pair data with insert sizes of 5kb and 8kb in a 5kb and 8kb sliding windows, respectively. On average, our final genome assembly was supported by 59.57% and 58.37% mate-pair reads from the 5-kb and 8-kb libraries. The complete mt genome sequence was deposited to GenBank (accession number KX028885).

### Sequence annotation

We aligned our assembly to the mt genes from 18 representative land plants with BLAST (identity > = 80% and E-value < = 1e-5) and identified ORFs using ORF finder (http://www.ncbi.nlm.nih.gov/gorf/gorf.html) [4]. Introns were depicted by using Rfam (v1.1 with default parameters, http://rfam.xfam.org) [42] (S1 Table) and tRNA genes were identified by using BLAST (v2.2.26+) and tRNAscan-SE (v1.23) [43]. All rRNA genes were identified similarly. The cp-derived regions were identified by comparing mt genome with cp genome (GenBank accession number KX028884) based on BLAST (identity > = 80%, E-value < = 1e-5 and length > = 50bp). REPuter and tandem repeat finder were used to identify forward, palindromic, and tandem repeats (https://bibiserv2.cebitec.uni-bielefeld.de/reputer and http://tandem.bu.edu/trf/trf.html) [44, 45].

### Sequence variants

Sequence variants were identified based on HiSeq paired-end data with 180-bp insert size. The raw reads were mapped to the final mt genome by using bowtie2 (version 2.2.4) [36], and the variants were called by using RGAAT tool, which developed in our laboratory (https://sourceforge.net/projects/rgaat/), and samtools and bcftools (version 1.2) [37–39]. To eliminate false positives, we only kept the variations identified by both methods. To evaluate the variations between the two palm species, *C*. *nucifera* and *P*. *dactylifera*, MUMmer3 was used for genome alignment [46].

### RNA editing analysis

We predicted putative RNA editing sites based on 8 public RNA-Seq datasets of coconut palm (SRR1063404, SRR1063407, SRR1137438, SRR1173229, SRR1265939, SRR1273070, SRR1273180, and SRR606452). After filtering the low quality reads and removing the adapter sequences by Trimmomatic (version 0.33) [47], we mapped all high-quality reads to the mt genome using GSNAP (version 2014-12-19) with the options “-N 1 and -force-xs-dir” (all other options are default) [48]. The candidate RNA editing loci were filtered through read mapping with the following criteria: (1) there are more than 2 aligned reads for each alternative allele, and (2) the percentage of the alternative allele must be equal or above 50%. We identified 845 RNA editing sites using REDO tool (https://sourceforge.net/projects/redo/) and predicted putative RNA editing sites in protein-coding genes using the web-based PREP-mt program with cutoff score 0.6 (http://prep.unl.edu/) [49].

### Phylogenetic analysis

Thirty-one representative mt protein coding genes were extracted from 19 species, including 8 monocots, 6 eudicots, and one each from gymnosperm (*Cycas taitungensis*), vascular plant (*Phlegmariurus squarrosus*), liverwort (*M*. *polymorpha*), hornwort (*Phaeoceros laevis*), and moss (*Physcomitrella patens*). Their amino acid sequences were aligned by using clustalw2 (version 2.1) [50]. We used both statistical method, Maximum Likelihood (ML) with Jones-Taylor-Thornton (JTT) substitution model and Maximum Parsimony (MP) in MEGA (version 6.06) for phylogenies of concatenated aligned sequences with 1000 bootstrap [51]. The gaps or missing data were eliminated when the site coverage below 90%. Phylogenetic trees were visualized with EvolView program [52].

### Transcriptome analysis

We counted the number of reads for each gene for mt genome using an in-house Perl script and identified differentially expressed genes using DESeq (version 1.20.0) [53]. For identifying the novel genes, we used Trinity (version 2.0.6) to construct transcripts based on GSNAP mapping results [54]. If different mt genes were assembled into one sequence, we assigned them to polycistronic transcription unit.

## Results and Discussion

### The *C*. *nucifera* mt genome content

**Fig 1:**
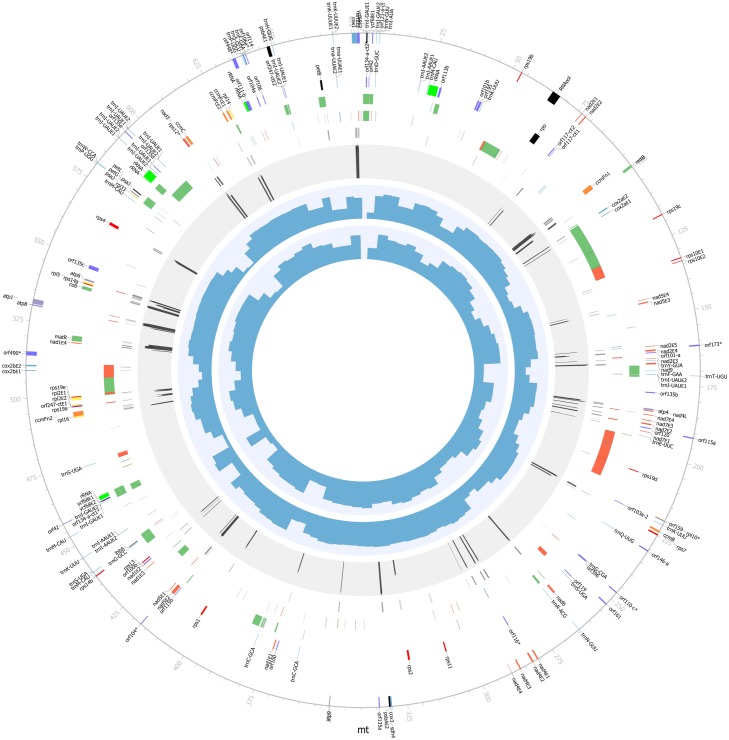
Circular display of *C. nucifera* mt genome. We display (from outside to inside): physical map scaled in kb; coding sequences transcribed in the clockwise and counterclockwise directions (*nad* in red; *cob*, *matR* and *mttB* in green; *cox* in blue; *atp* in purple; *ccm* in orange; *rpl* in yellow; *rps* in dark red; rRNA in dark green; tRNA in dark blue; *orf* in dark purple; and others in black); chloroplast-derived regions (green); repeats (forward repeats in green, palindrome repeats in red and tandem repeats in blue); RNA edit sites (synonymous in green and non-synonymous in red); gene conserve scores (black); proper HiSeq mate-pair (MP) reads percent with insert size 5kb and 8kb (blue); and the four regions (thick lines indicate IRs and thin lines indicate LSC and SSC). * indicates pseudogenes.

**Fig 2:**
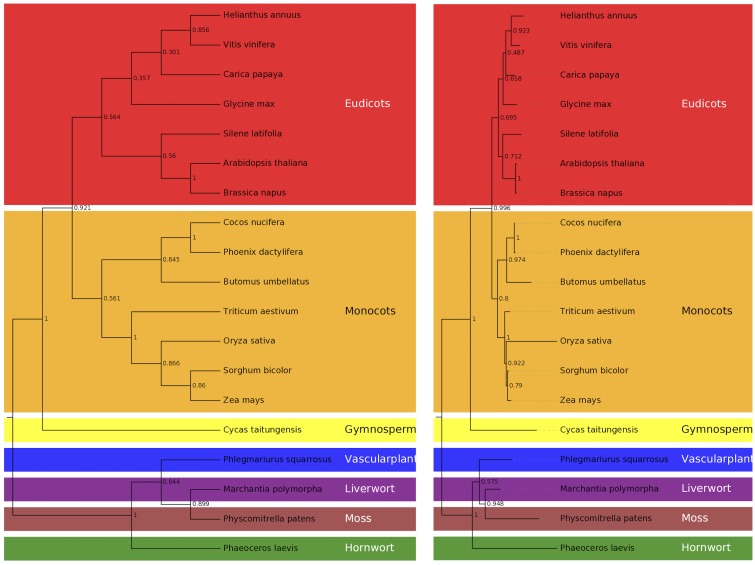
Phylogenetic trees of 31 mt proteins from 19 plant species. Shown in the left is a maximum parsimony tree and the right is a maximum likelihood tree based on MEGA 6.06. The *C*. *nucifera* mt proteins form a cluster with those of *P*. *dactylifera* and *B*. *umbellatus* among monocotyledons.

We started *C*. *nucifera* (Oman local Tall variety) mt genome assembly based solely on the Roche/454 GS FLX data, including 7,617,799 single reads, 2,884,708 paired reads with 3-kb insert size, and 1,594,036 paired reads with 8-kb insert size. After homopolymer correction using the Illumina reads, we have an assembly of 678,653bp in length (Fig 1; see Materials and Methods). It encodes 72 proteins (87 protein-coding genes, 8.62% of mt genome), 9 truncated proteins (codon frameshift mutations; 10 pseudogenes, 0.83% of mt genome), 23 tRNAs (corresponding to 17 amino acid codons and one stop codon, 42 tRNA-coding genes, 0.46% of mt genome), and 3 ribosomal RNAs (6 rRNA-coding genes, 1.51% of mt genome), which all together constitute a gene content of 11.43% (77,542bp) (Table 1). Among them, 13 proteins (15 protein-coding genes), 2 truncated proteins (codon frameshift; 3 pseudogenes), 11 tRNAs (corresponding to 10 amino acid codons, 13 tRNA-coding genes) and 3 ribosomal RNAs (3 rRNA-coding genes) locate in the chloroplast-derived regions, which are accounted for 5.07% of the genome sequence. The GC contents of protein-coding genes, pseudogenes, tRNAs, rRNAs, and the remaining non-coding sequences are 44.5% (58,895bp), 47.7% (5,294bp), 41.1% (3,092bp), 53.5% (10,261bp), and 45.5% (601,111bp), respectively. The genome harbors 0.49% tandem (3,310bp) and 17.26% long repeats (≥100bp). In addition, there are 13 co-transcribed gene clusters, including conserved *18S-5S rRNA* and *nad3-rps12* among angiosperm mt genomes [55]. Our phylogenetic analysis shows that *C*. *nucifera* clusters with *P*. *dactylifera* and *Butomus umbellatus* among the monocotyledon plants (Fig 2).

**Table 1:** The gene content of the *C*. *nucifera* mt genome.

Function	Genes
Genes of Mitochondrial Origin (109/85)	
Complex I (9)	*nad1*, *nad2*, *nad3*, *nad4*, *nad4L*, *nad5*, *nad6*, *nad7*, *nad9*
Complex II (1)	*sdh4*
Complex III (1)	*Cob*
Complex IV (4/3)	*cox1*, *cox2a*, *cox2b*, *cox3*
Complex V (5)	*atp1*, *atp4*, *atp6*, *atp8*, *atp9*
Cytochrome c biogenesis (5/4)	*ccmB*, *ccmC*, *ccmFc*, *ccmFn1*, *ccmFn2*
Ribosome large subunit (3)	*rpl2*, *rpl5*, *rpl16*
Ribosome small subunit (14/10)	*rps1*, *rps2*, *rps4*, *rps7*, *rps10*, *rps11*, rps12, *rps13*, *rps14a*, *rps19a*, *rps19b*, *rps19c*, *rps19d*, *rps19e*
Intron maturase (1)	*matR*
SecY-independent transporter (1)	*mttB*
rRNA genes (3)	*5sRNA*, *18sRNA*, *26sRNA*
tRNA genes (29/18)	*trnStop-UUA*, *trnC-GCA*, *trnD-GUC*, *trnE-UUC*, *trnF-GAA*, *trnG-CGA*, *trnI-AAU*(x2), *trnI-AUA*, *trnI-UAU*(x5), *trnK-UUU*(x4), *trnM-CAU*(x2), *trnN-GUU*, *trnP-UGG*(x2), *trnQ-UUG*, *trnS-GCU*, *trnS-UGA*(x2), *trnW-CCA*, *trnY-GUA*
Hypothetical genes (26/19)	*orf100*(x2), *orf101*(x2), *orf103*, *orf104a*, *orf106*, *orf111*, *orf114*, *orf115*(x2), *orf117*, *orf119*, *orf120*, *orf135*(x5), *orf146*, *orf159*, *orf161*, *orf195*, *orf222*, *orf247*, *orf396*
Pseudogenes (7)	*orf104*, *orf106a*, *orf110*, *orf116*, *orf173*, *orf448*, *orf490*
Genes of Chloroplast Origin (34/29)	
Functional genes (11/10)	*lhbA*, *petB*, *petG*, *petL*, *psaJ*, *psbA*, *rpl14*, *rpl33*, *rps14b*, *ycf68*(x2)
Hypothetical genes (4/3)	*orf42*(x2), *orf113*, *orf121*
rRNA genes (3)	*5sRNA*, *18sRNA*, *26sRNA*
tRNA genes (13/11)	*trnC-GCA*, *trnF-GAA*, *trnG-GCC*, *trnH-GUG*, *trnI-GAU*(x2), *trnI-UAU*, *trnM-CAU*(x2), *trnN-GUU*, *trnR-ACG*, *trnS-UGA*, *trnT-UGU*
Pseudogenes (3/2)	*rpl10*, *orf134*(x2)
Genes of Nuclear Origin (2): *rpo*, *RNA_pol*	

Note: The two numbers in parentheses after the item of the first column stand for total and unique genes; the number in parentheses after gene name is gene copy number.

### Protein-coding, rRNA, and tRNA genes

The *C*. *nucifera* mt genome encodes 50 known functional and 22 hypothetical proteins. Among the first group, 23 proteins are related to the electron transport chain, including 9 subunits of nicotinamide adenine dinucleotide dehydrogenase (complex I), one subunit of succinate dehydrogenase (complex II), one apocytochrome b (complex III), 3 subunits of cytochrome c oxidase (complex IV), 5 subunits of ATP synthase F1 (complex V), and 4 cytochrome c biogenesis proteins (Table 1).

First, when compared the *C*. *nucifera* mt proteins to 18 other plants (S1 Table and S1 Fig), we identified *sdh* gene that is unique to the coconut and absent in 7 other monocots. Second, similar in the cases of *Vitis vinifera*, *S*. *latifolia*, and *P*. *dactylifera*, RNA polymerase genes are identified in the mt genome (one RNA polymerase and one DNA-dependent RNA polymerase). Third, the *C*. *nucifera* mt genome has the highest copy number of *rps19* genes (5 copies) in all 19 inspected species, followed by *V*. *vinifera* (3 copies). Fourth, there is no *rps3* gene in *C*. *nucifera* mt genome, whereas it exists in 7 other monocot species. Fifth, *rpl10* (pseudogene) and *rps11* (protein-coding gene) are found only in *P*. *dactylifera* and *C*. *nucifera* among all 8 monocots. Last, a few of cp-derived genes are identified in this genome, including 15 protein-coding (such as *rpl14*, *rpl33* and *rps14*), 3 rRNA, and 13 tRNA genes as well as 3 pseudogenes.

The mt genome contains 42 tRNA genes; 12 of them have introns (9 mt tRNAs and 3 cp-derived tRNAs) (Table 2). Among these tRNA genes, all correspond to 17 amino acids but are absent for the rest three: Ala, Leu, and Val. The tRNA genes for amino acids Thr, His, Arg, Gly, and tRNA^Ile^(GAU) are only found in the cp-derived regions. These results are consistent with previous studies that the mt tRNA genes are replaced by those of the cp-derived tRNA gradually [24, 56].

**Table 2:** Codon usage and codon-anticodon recognition pattern in the *C*. *nucifera* mt genome.

AA	C	No.	R	tRNA^a^	AA	C	No.	R	tRNA	AA	C	No.	R	tRNA	AA	C	No.	R	tRNA
Phe	UUU	520	1.11		Ser	UCU	370	1.41		Tyr	UAU	349	1.31	AUA	Cys	UGU	140	1.06	
	UUC	416	0.89	GAA2*		UCC	275	1.05			UAC	184	0.69	GUA		UGC	125	0.94	GCA2*
Leu	UUA	332	1.21			UCA	276	1.05	UGA3*	Ter	UAA	30	1.17		Ter	UGA	20	0.78	
	UUG	344	1.25			UCG	219	0.84	CGA		UAG	27	1.05		Trp	UGG	258	1.00	CCA
	CUU	334	1.21		Pro	CCU	323	1.29		His	CAU	307	1.35		Arg	CGU	224	1.16	ACG*
	CUC	232	0.84			CCC	201	0.80			CAC	149	0.65	GUG*		CGC	134	0.69	
	CUA	230	0.84			CCA	327	1.30	UGG2	Gln	CAA	317	1.33	UUG		CGA	256	1.32	
	CUG	179	0.65			CCG	153	0.61			CAG	158	0.67			CGG	161	0.83	
Ile	AUU	462	1.17	AAU2	Thr	ACU	283	1.29		Asn	AAU	348	1.24		Ser	AGU	236	0.90	
	AUC	394	1.00	GAU2**		ACC	239	1.09			AAC	215	0.76	GUU2*		AGC	194	0.74	GCU
	AUA	330	0.83	UAU6*		ACA	209	0.96	UGU*	Lys	AAA	463	1.04	UUU4	Arg	AGA	301	1.21	
Met	AUG	429	1.00	CAU4**		ACG	144	0.66			AAG	425	0.96			AGG	197	0.79	
Val	GUU	303	1.20		Ala	GCU	414	1.58		Asp	GAU	410	1.29		Gly	GGU	355	1.23	
	GUC	207	0.82			GCC	227	0.86			GAC	226	0.71	GUC		GGC	172	0.59	GCC*
	GUA	279	1.11			GCA	252	0.96		Glu	GAA	486	1.21	UUC		GGA	387	1.34	
	GUG	220	0.87			GCG	158	0.60			GAG	320	0.79			GGG	243	0.84	

Note: AA, Amino acid; C, Codon; R, relative synonymous codon usage;

^a^, the content of tRNA including anticodon and tRNA; the cp-derived tRNA is indicated with asterisks (*).

### Cp-derived regions, introns, and repeats

The plant cp and mt genomes are known to have extensive and widespread homologies due to sequence transfer [57, 58]. The transfer of cp genomic DNA to that of the mt genome has been going on for at least 300 million years [59]. In the *C*. *nucifera* mt genome, there are 33 cp-derived regions with a length range of 64 to 3,365bp (S2 Table). The total length of cp-derived regions is 34,395bp and the coding region is 37.58% (12,925bp), which is higher than mt gene content (11.41%) but lower than cp gene content (61.17%). The GC content of the cp-derived regions is 41.9%, which is between those of the cp (37.44%) and mt (45.50%) genomes. A similar trend is found in *P*. *dactylifera* with GC contents of 37.23%, 37.40%, and 45.1% for cp, cp-derived region, and mt DNA, respectively [24, 60]. These results suggest that cp-derived sequences, to some extent, have evolved to be close to the mt genome sequences in GC contents and gene coding fractions after being transferred into mt genomes.

In the *C*. *nucifera* mt genome, there are 28 intron-containing genes (16 protein-coding genes and 12 tRNA genes), and according to the prediction based on Rfam, one group I intron (not located in gene regions) and 23 group II introns were identified. Among 23 group II introns, 15 locate in 8 protein-coding genes (*nad1*, *nad2*, *nad4*, *nad5*, *nad7*, *rps10*, *cox2a* and *cox2b*) and 2 are in 2 tRNA genes (two *trnI-GAU*). Although mitochondrial tRNA genes do not possess introns in general, we identified 12 intron-containing tRNA genes (including 3 cp-derived tRNA genes) in the assembly. Among 18 other plants (S1 Table), *M*. *polymorpha* (liverwort), *C*. *taitungensis* (gymnosperm), *B*. *umbellatus* (monocot), *P*. *dactylifera* (monocot), *Zea mays* (monocot), and *V*. *vinifera* (eudicot) have one (*tRNA-Ser*), one (*tRNA-Val*), two (*tRNA-Ile* and *tRNA-Ala*), three (*tRNA-Lys*, *tRNA-Asn* and *tRNA-Stop*), three (*tRNA-Leu*/pseudo, *tRNA-Leu*/pseudo and *tRNA-Ile*), and one (*tRNA-Lys*) intron-containing tRNA genes, respectively. It shows that the *C*. *nucifera* mt genome has the largest intron-containing tRNA genes among all analyzed sequences.

The *C*. *nucifera* mt genome contains 0.49% tandem repeats, which are compatible with those of *P*. *dactylifera* (0.33%) (S3 Table). However, it harbors 17.26% long repeats (> = 100bp), and the number is significantly higher than that of *P*. *dactylifera* (2.3%) but compatible with those of other monocot species, such as *Triticum aestivum* (15.9%), *Sorghum bicolor* (16.2%), and *Zea may* (19.1%) (S4 Table).

### Sequence variation analysis

Based on the HiSeq data, we identified 202 and 157 variations in different places of the genome, using samtools & bcftools and RGAAT (https://sourceforge.net/projects/rgaat/), respectively; among the total, 102 variations are cross-discovered based on both methods (S5 Table). To reduce false positives, we only used the 102 shared variations (100 SNPs and 2 insertions) for further analysis. First, 48 out of the total are found in the cp-derived regions. Among all variations, only 5 SNPs are in the protein-coding genes, including 3 synonymous SNPs of *rps1*, *rps2*, and *rpl14* (cp-derived) and two non-synonymous SNPs of *orf247-ct* (S6 Table). Other 6 SNPs and 1 insertion are found in 5 tRNA genes, whereas the remaining 89 SNPs and 1 insertion are non-coding. Second, according to the variation types, there are 23 transitions (Ti) and 77 transversions (Tv), leading to a Ti/Tv ratio of 0.30. If we remove the cp-derived regions from the analysis, the ratio becomes 0.06 (Ti/Tv ratio; 3 Ti and 50 Tv SNPs). It is in sharp contrast to that of the nuclear genome, where the ratios range ~2.0–2.1 in genome-wide and 3.0–3.3 in exonic sequences [61, 62]. The Ti/Tv ratio in the coconut mt genome is much lower than what is in the nuclear genome, as well as a random prediction (0.5). It supports the observation that DNA replication and repair mechanisms are very different between mt and nuclear genomes. Third, we further scrutinized the data to exclude other possibilities that may lead to biased results. According to the Roche/454 and Illumina sequence coverage, there are ~2x, ~42x, and ~235x of the Roche/454 reads, as well as ~20x, ~1788x, and ~6000x of the Illumina reads for nuclear, mt, and cp DNA, respectively, which reflect a copy number ratio among them as ~1:55:209 on average. This result indicates that only 1.79% reads of similar sequences may be an origin of the nuclear genome in the mt genome datasets, which can be excluded readily during sequence variation identification (alternative allele proportion> = 15%). Similarly, for the cp-derived regions, sequence variations are more likely from cp (79.17%) rather than from the nuclear or mt genomes.

Comparing to the two taxonomically closest species *P*. *dactylifera* and *B*. *umbellatus* in this study, we only aligned 54.45% and 14.15% of the *C*. *nucifera* mt genome, respectively, using bl2seq (S2 Fig) [63]. To further evaluate mt genome variations between the two palm species *P*. *dactylifera* and *C*. *nucifera*, we used MUMmer to compare the alignable regions and identified 2,442 SNPs and 1,122 indels, coming up with an average rate of 5 variations per 1,000bp (S3 Fig).

### RNA editing

RNA editing is universal to almost all plant mt transcripts [64, 65] with features of tissue specific and partial edits [66]. Different species have different RNA editing sites and the number of RNA editing sites ranges from 200 to 600 in angiosperm[67]. The public RNA-Seq data in NCBI are excellent and untapped resources, where we found 8 RNA-Seq datasets from coconut (two of Tall cultivars and 6 of Dwarf cultivars) for our RNA editing analysis [68]. To differentiate sequencing errors and SNPs from editing, we only kept the RNA editing sites with more than 2 supporting reads and with at least 50% edited reads. The criteria lead to the identification of 845 RNA editing sites in 56 protein-coding genes and 36 RNA editing sites are in the cp-derived regions (S7 Table). Among the total RNA editing sites (92 synonymous and 753 nonsynonymous), there are 811 C->T, 26 G->A and 8 T->C sites. We compared tissue disparity among the 8 samples, where healthy leaf1 has the most RNA editing sites (697, 82.49%, 18 unique) and embryo has the least RNA editing sites (489, 57.87%, 22 unique). In addition, 297 RNA editing sites are shared by all 8 samples. Since the 8 samples are from two cultivars, we partitioned the editing sites between the Dwarf and Tall cultivars, yielding 835 and 675 RNA editing sites, respectively, unique to each cultivar and 665 shared. Considering the codon changing edits, we ranked the top six codon changes: TCA->TTA (95, 11.24%), TCT->TTT (67, 7.93%), TCG->TTG (58, 6.86%), CCA->CTA (50, 5.92%), TCC->TTC (45, 5.33%), and CGG->TGG (45, 5.33%); 5 of them changed the second codon position. Moreover, the top six edited codons are TTT (135, 15.98%), TTA (118, 13.96%), TTG (72, 8.52%), TTC (58, 6.86%), CTA (51, 6.04%), and CTT (50, 5.92%).

We also predicted 648 RNA editing sites using PREP-mt program in 45 genes. Comparing the RNA editing sites identified by using the two methods, we have 591 shared, 57 unique to PREP-mt program, and 212 unique to our method; the underestimation of PREP-mt program becomes obvious.

### Gene expression analysis based on transcriptome data

**Fig 3:**
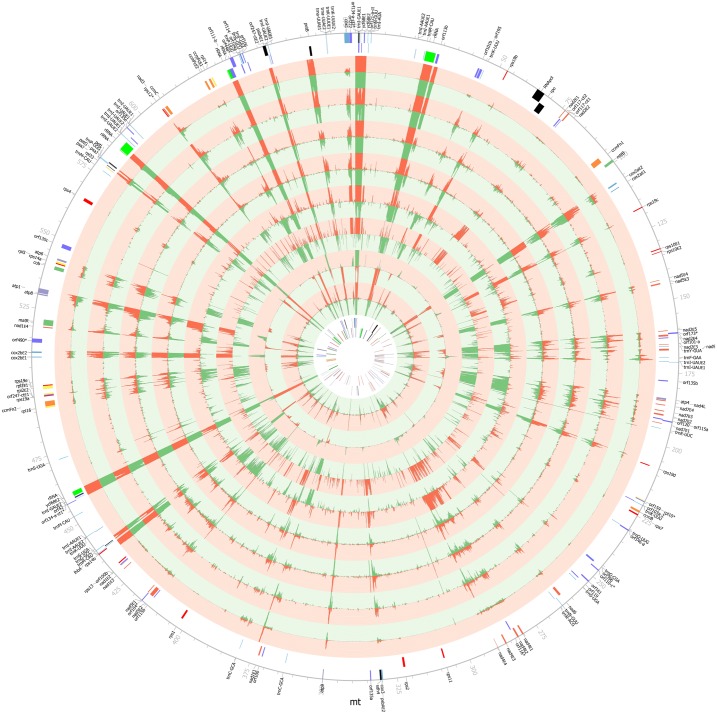
Circular display of *C*. *nucifera* mt transcriptomes. We display (from outside to inside): physical map scaled in kb; coding sequences transcribed in the clockwise and counterclockwise directions (*nad* in red; *cob*, *matR* and *mttB* in green; *cox* in blue; *atp* in purple; *ccm* in orange; *rpl* in yellow; *rps* in dark red; rRNA in dark green; tRNA in dark blue; *orf* in dark purple; and others in black); histogram of transcriptome data (plus strand in red and minus strand in green, standing for normalized average coverage value per 100 bp ranging from 0 to 100) for sample Health_leaf1, CYD_leaf, Callus, RWDS_leaf, Endosperm, Embryo, Health_leaf2 and Leaf_fruit; coding sequences transcribed in the clockwise and counterclockwise directions; and the four regions (thick lines indicate IRs and thin lines indicate LSC and SSC). * indicates pseudogene.

Using the RNA-Seq datasets, we obtained mt transcriptomic profiles for the 8 samples (Fig 3 and Table 3). Three healthy leaf samples have the most abundant mapped reads (3.71% to 1.47%), two disease related leaf samples and embryogenic callus fall into the second abundance group (0.29% to 0.24%), whereas endosperm and embryo are the least abundant (0.12% and 0.05%, respectively). Read abundance of mt sequence coincides with tissue characteristics but read coverage shows a different pattern. First, root wilt disease susceptible (RWDS) leaf has the highest read coverage (71.92%) and coconut yellow decline (CYD) leaf has the lowest read coverage (34.94%). Second, healthy leaf samples (54.77% to 68.00%) and embryogenic callus (57.52%) have higher read coverage as opposed to embryo (37.34%) and endosperm (45.28%) (Table 4).

**Table 3:** Mt transcriptome profiles of the 8 coconut RNA-Seq datasets.

Cultivar	Tissue	SRA accession No.	Length	Original fragments	High quality fragments	Percent	mt mapping fragments	mt mapping percent^a^
Malayan Red Dwarf	Healthy_leaf1	SRR1063404	202	36,009,632	32,555,041	90.41%	1,337,565	3.71%
Malayan Red Dwarf	CYD_leaf (CYD-infected leaf)	SRR1063407	202	35,467,948	32,141,745	90.62%	101,295	0.29%
West Coast Tall	Callus (Embryogenic callus)	SRR1137438	152	50,839,994	42,267,444	83.14%	121,356	0.24%
Chowghat Green Dwarf	RWDS_leaf (root wilt disease susceptible leaf)	SRR1173229	202	119,333,177	113,394,045	95.02%	289,707	0.24%
Dwarf	Endosperm	SRR1265939	202	51,540,183	48,892,847	94.86%	60,531	0.12%
Dwarf	Embryo	SRR1273070	337	40,564,276	37,752,443	93.07%	21,021	0.05%
Dwarf	Healthy_leaf2 (Young leaf)	SRR1273180	252	60,030,680	54,291,251	90.44%	882,592	1.47%
Hainan Tall	Leaf_fruit (Spear leaf, young leaf and fruit flesh)	SRR606452	180	27,465,703	27,063,513	98.54%	447,384	1.63%

Note: CYD, coconut yellow decline;

^a^, the percent is corresponding to high quality fragments.

**Table 4:** The mt read coverage of the 8 coconut RNA-Seq datasets.

Coverage	Type	Health_leaf1	CYD_leaf	Callus	RWDS_leaf	Endosperm	Embryo	Health_leaf2	Leaf_fruit
0	Bases	306960	441453	288308	190574	371400	425207	285658	217158
	Percent	45.23%	65.05%	42.48%	28.08%	54.73%	62.65%	42.09%	32.00%
1–4	Bases	198091	137894	253794	276303	156258	145853	226692	242753
	Percent	29.19%	20.32%	37.40%	40.71%	23.02%	21.49%	33.40%	35.77%
5–9	Bases	39147	35099	52538	70598	53185	42874	45553	61987
	Percent	5.77%	5.17%	7.74%	10.40%	7.84%	6.32%	6.71%	9.13%
10–99	Bases	98440	50247	70713	114064	83923	52614	89257	113677
	Percent	14.51%	7.40%	10.42%	16.81%	12.37%	7.75%	13.15%	16.75%
100–999	Bases	20793	10058	7332	17646	12001	12088	15898	32165
	Percent	3.06%	1.48%	1.08%	2.60%	1.77%	1.78%	2.34%	4.74%
> = 1000	Bases	15222	3902	5968	9468	1886	17	15595	10913
	Percent	2.24%	0.57%	0.88%	1.40%	0.28%	0.00%	2.30%	1.61%
> = 1	Percent	54.77%	34.94%	57.52%	71.92%	45.28%	37.34%	57.90%	68.00%

There are 113 out of the total 145 genes expressed in at least two samples whereas only 3 genes (*rpo*, *trna-UUA*, and *trnI-AAU*) expressed in one sample (Young_leaf) (S8 Table). The number of expressed genes is consistent with read coverage. CYD leaf has the least expressed genes (92) as opposed to RWDS leaf that has the most (116). The genes *petL* and *orf247-ct*, which have stop codon in the middle of gene sequence, are highly expressed, however, we have not found any RNA editing sites to rescue the normal protein-coding function. Both of them need to be validated in future studies. All pseudogenes have relatively high expression level in all samples other than *rpl10*. According to transcriptomic profiles, we found 13 polycistronic transcripts among 37 genes (S9 Table). The conservative co-transcribed gene clusters *rps12*-*nad3* and *5SrRNA*-*18SrRNA* are also found in our mt genome.

**Fig 4:**
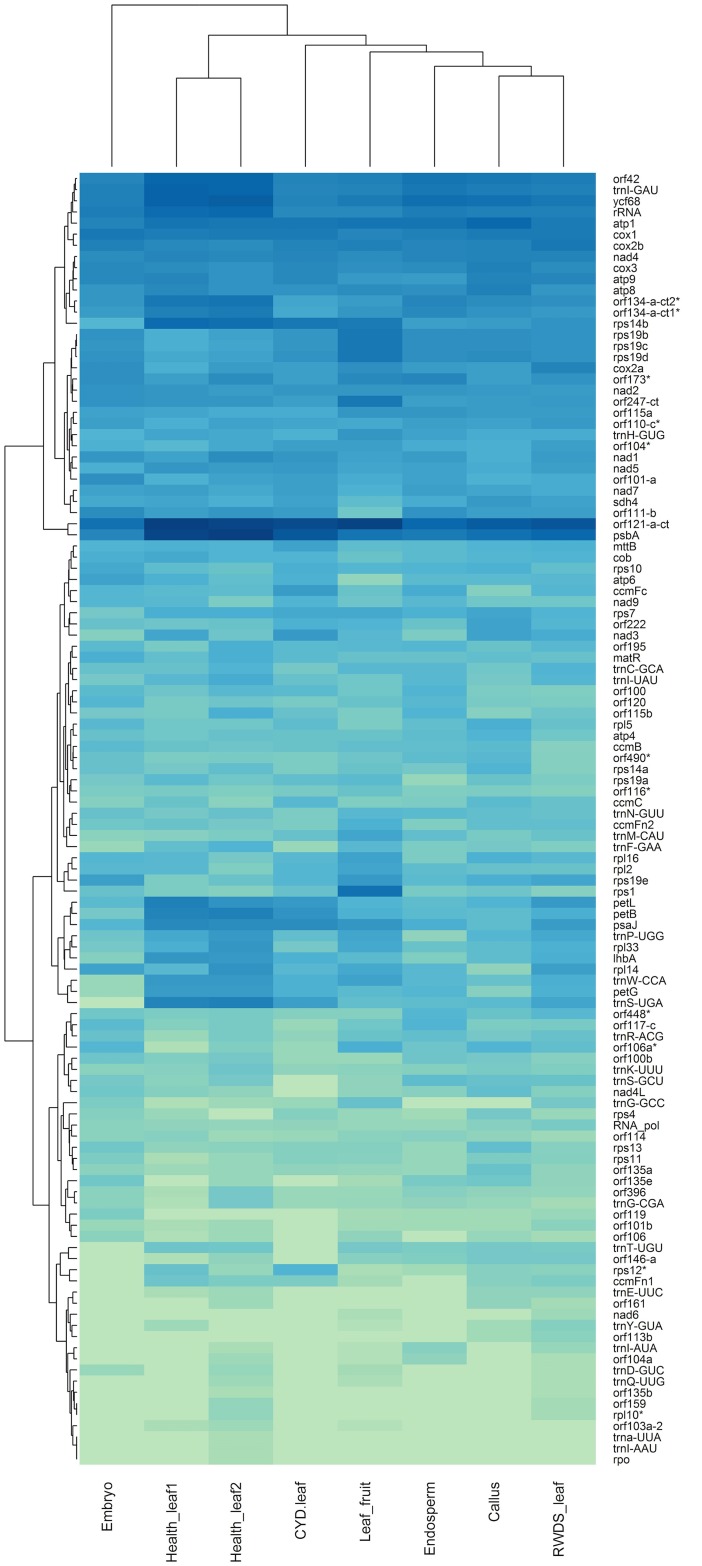
Expression patterns of mt genes among 8 RNA-Seq datasets. The expression levels are normalized based on DEseq.

According to the gene expression profiles (Fig 4), we have observed several obvious features. First, the genes can be divided into three categories according to expression intensity: highly, moderately, and lowly expressed. Second, among 33 highly expressed genes, there are only two tRNA genes (*trnI-GAU* and *trnH-GUG*). Third, three of the five *rps19* copies are highly expressed and the rest are moderately expressed. Fourth, only *nad6* and *ccmFn1* of the 25 respiration related genes are lowly expressed.

### Phylogenetic relationships

Our phylogenetic trees are built based on 31 mt protein-coding genes from 19 selected plants (8 monocots and 6 eudicots, as well as one each of gymnosperm, vascular plant, liverwort, hornwort, and moss; Fig 2). The maximum-likelihood (ML) tree has higher bootstrap values than the maximum parsimony (MP) tree except for the node of *S*. *bicolor* and *Z*. *mays* and the node between *P*. *squarrosus* and the group of *M*. *polymorpha* and *P*. *patens*. Most nodes have bootstrap values larger than 65% except for one node (49%) among *Helianthus annuus*, *V*. *vinifera* and *Carica papaya* and another node (58%) between *P*. *squarrosus* and the group of *M*. *polymorpha* and *P*. *patens* from ML method. Both methods have high bootstrap values (97% and 85%) for subgroup of *C*. *nucifera*, *P*. *dactylifera* and *B*. *umbellatus* in monocots. Previous studies indicate that date palm appears to be the most basal among monocots [24, 69]. Moreover, date palm has certain miRNA families only found in eudicots [70]. Taken together, these results suggest that Arecaceae separated from the monocotyledon clade earlier than other plant families.

## Conclusion

Despite the fact that the *C*. *nucifera* mt genome is as large as 678,653bp in length, we have assembled it using a variety of datasets and information, including all plant mt genome sequences, *C*. *nucifera* mt sequence datasets from different platforms and libraries with variable insert sizes, and specialized bioinformatics tools suitable for different purposes. The genome sequence variations and RNA editing sites based on transcriptomic data are all invaluable for further biological studies. Phylogenetic analysis indicates that Arecaceae separated from the rest of monocotyledons earlier in flowering plant evolution.

## Supporting Information

S1 Fig The homologous mt genes among *C*. *nucifera* and 18 other representative plant species. (TIF)

S2 Fig A mt genome comparison among *C*. *nucifera*, *P*. *dactylifera* and *B*. *umbellatus*. (TIF)

S3 Fig Palm mt and cp genome comparisons between *P*. *dactylifera* and *C*. *nucifera* (Ref) based on MUMmer. (A) mt genomes and (B) cp genomes. Unlike the cp genomes, variations between the mt genomes are much higher.
(TIF)

S1 Table The homologous mt genes among *C*. *nucifera* and 18 other representative species. (DOCX)

S2 Table The cp-derived regions in the *C*. *nucifera* mt genome. (DOCX)

S3 Table Tandem repeats identified in the *C*. *nucifera* mt genome by using Tandem Repeats Finder. (XLSX)

S4 Table Long repeats (> = 100bp, forward and palindrome) identified in the *C*. *nucifera* mt genome based on REPuter. (XLSX)

S5 Table The common variations identified in the *C*. *nucifera* by using samtools & bcftools and RGAAT. (XLSX)

S6 Table The functional evaluation of common variations in the *C*. *nucifera* mt genome. (XLSX)

S7 Table RNA editing sites identified by using RNA-Seq data and PREP-mt program. NT, nucleotide; AA, amino acid.
(XLSX)

S8 Table Reads of mt genes in the 8 coconut RNA-Seq datasets. (XLSX)

S9 Table 13 polycistronic transcripts identified based on the 8 coconut RNA-Seq datasets. (DOCX)
