# Supplementary figures and images for: Complete Sequence and Analysis of Coconut Palm (Cocos nucifera) Mitochondrial Genome
Source: PLoS One. 2016 Oct 13;11(10):e0163990. doi: 10.1371/journal.pone.0163990 (PMC5063475; doi:10.1371/journal.pone.0163990)

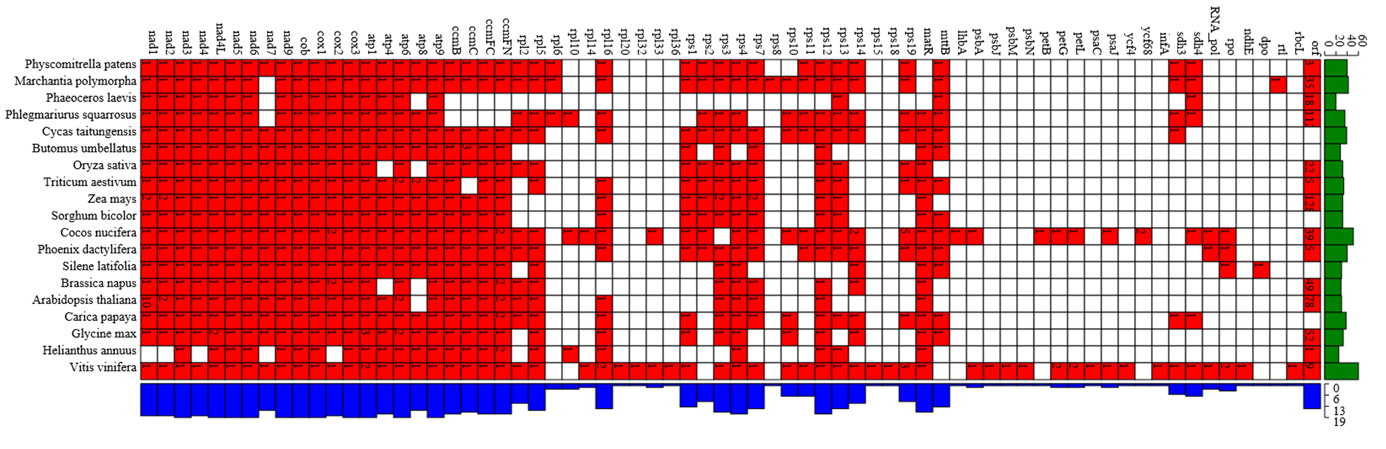

Supplement: S1 Fig — (TIF) [file pone.0163990.s001.tif]

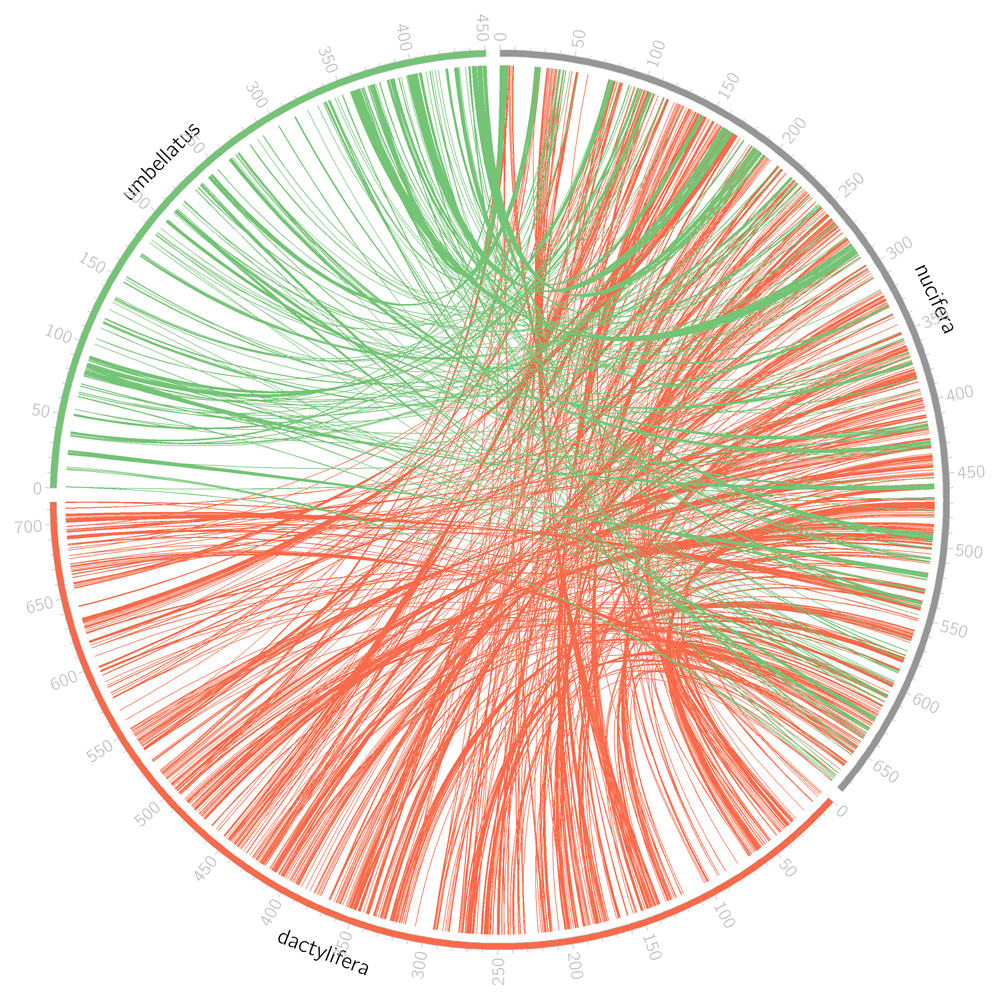

Supplement: S2 Fig — (TIF) [file pone.0163990.s002.tif]

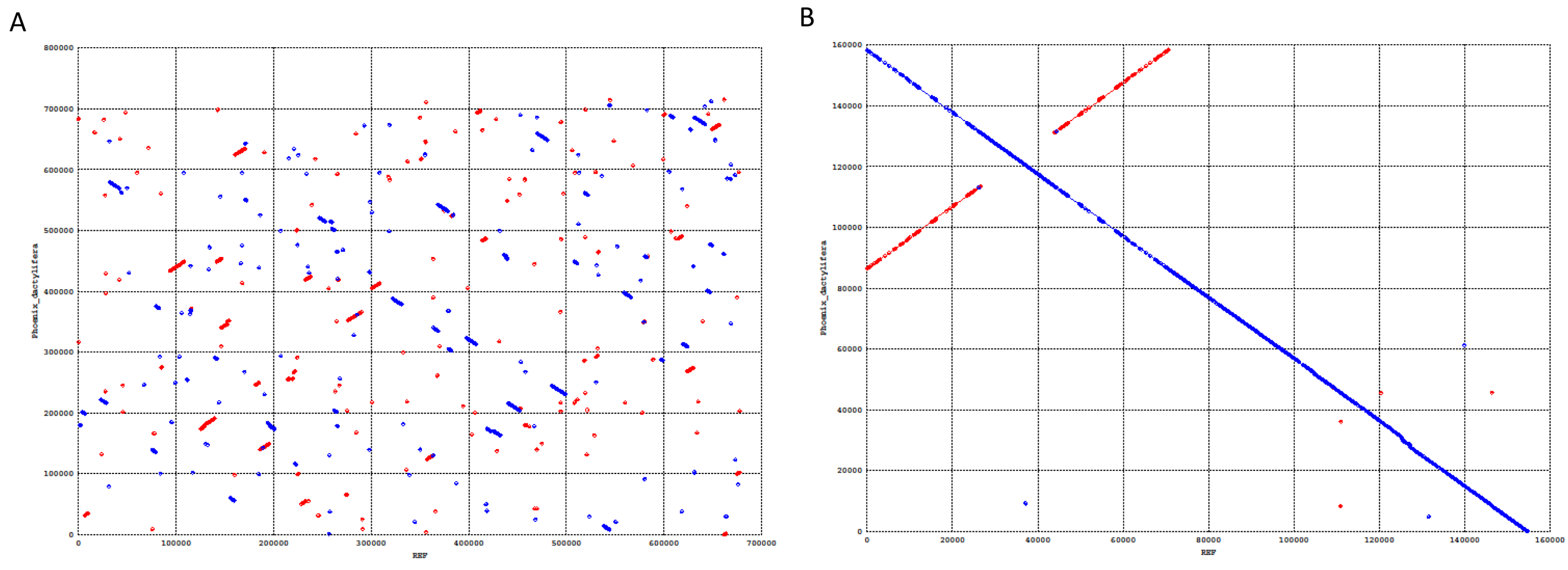

Supplement: S3 Fig — (A) mt genomes and (B) cp genomes. Unlike the cp genomes, variations between the mt genomes are much higher. (TIF) [file pone.0163990.s003.tif]
