# Supplementary material for: Complete Sequence and Analysis of Coconut Palm (Cocos nucifera) Mitochondrial Genome
Source: PLoS One. 2016 Oct 13;11(10):e0163990. doi: 10.1371/journal.pone.0163990 (PMC5063475; doi:10.1371/journal.pone.0163990)
Supplement: S2 Table — (DOCX) [file pone.0163990.s005.docx]

**S2 Table. The cp-derived regions in the *C. nucifera* mt genome**

| **Chrom** | **Start** | **End** | **Length** | **Gene** | **Gene_length*** |
| --- | --- | --- | --- | --- | --- |
| mt | 1 | 2546 | 2546 | *trnI-GAU*, *orf134-a-ct2*, *ycf68*, *orf42* | 985 |
| mt | 2648 | 3948 | 1301 | *orf121-a-ct* | 297 |
| mt | 27394 | 27490 | 97 |  |  |
| mt | 28614 | 30283 | 1670 | *orf113b* | 744 |
| mt | 152479 | 152590 | 112 |  |  |
| mt | 167807 | 171103 | 3297 | *trnF-GAA* | 73 |
| mt | 171234 | 172202 | 969 | *trnI-UAU*, *trnT-UGU* | 152 |
| mt | 186148 | 186211 | 64 |  |  |
| mt | 195434 | 195582 | 149 |  |  |
| mt | 261797 | 261970 | 174 |  |  |
| mt | 264230 | 264984 | 755 | *trnN-GUU*, *trnR-ACG* | 146 |
| mt | 264984 | 265089 | 106 |  |  |
| mt | 300551 | 300716 | 166 |  |  |
| mt | 363170 | 363256 | 87 |  |  |
| mt | 374204 | 374701 | 498 |  |  |
| mt | 379248 | 379336 | 89 |  |  |
| mt | 379469 | 379812 | 344 |  |  |
| mt | 380028 | 380277 | 250 | *trnC-GCA* | 71 |
| mt | 380383 | 380630 | 248 |  |  |
| mt | 381233 | 384091 | 2859 |  |  |
| mt | 414018 | 414340 | 323 |  |  |
| mt | 435956 | 438852 | 2897 | *rps14b*, *trnM-CAU*, *trnG-GCC* | 448 |
| mt | 438916 | 440364 | 1449 | *lhbA*, *trnS-UGA* | 282 |
| mt | 453111 | 453187 | 77 | *trnM-CAU* | 73 |
| mt | 457511 | 460875 | 3365 | *orf42*, *trnI-GAU*, *orf134-a-ct1*, *ycf68*, *18SrRNA* | 2482 |
| mt | 579131 | 582222 | 3092 | *rpl33*, *psaJ*, *trnP-UGG*, *trnW-CCA*, *petG*, *petL* | 791 |
| mt | 586525 | 589014 | 2490 | *5SrRNA*, *28SrRNA* | 3296 |
| mt | 626941 | 627081 | 141 |  |  |
| mt | 627115 | 627565 | 451 | *rpl14* | 444 |
| mt | 647218 | 648455 | 1238 | *psbA*, *trnI-UAU* | 1945 |
| mt | 648649 | 649158 | 510 |  |  |
| mt | 661068 | 661156 | 89 |  |  |
| mt | 661157 | 663648 | 2492 | *petB* | 696 |

Note: *: gene length is the total length of all genes. Sometimes, only one part of gene is located in cp-derived region, but we used gene full length because the other parts of gene are also coming from cp although the sequence changed too much for identification.
