# Supplementary material for: Complete Sequence and Analysis of Coconut Palm (Cocos nucifera) Mitochondrial Genome
Source: PLoS One. 2016 Oct 13;11(10):e0163990. doi: 10.1371/journal.pone.0163990 (PMC5063475; doi:10.1371/journal.pone.0163990)
Supplement: S9 Table — (DOCX) [file pone.0163990.s012.docx]

**S9 Table. 13 polycistronic transcripts identified based on the 8 coconut RNA-Seq datasets**

| **Strand** | **Start** | **End** | **Gene number** | **Gene list** |
| --- | --- | --- | --- | --- |
| - | 166024 | 167437 | 2 | *trnY-GUA*, *nad9* |
| - | 169850 | 171367 | 2 | *trnF-GAA*, *trnI-UAU* |
| + | 219495 | 219976 | 2 | *orf159*, *rpl10* |
| + | 330030 | 331016 | 3 | *cox3*, *psbA*, *sdh4* |
| + | 437922 | 439666 | 3 | *rps14b*, *trnM-CAU*, *trnS-UGA* |
| - | 438740 | 439217 | 2 | *trnG-GCC*, *lhbA* |
| - | 491342 | 499220 | 5 | *rpl16*, *ccmFn2*, *rps19a*, *rpl2*, *rps19e* |
| - | 579957 | 581292 | 3 | *psaJ*, *petG*, *petL* |
| + | 580502 | 580806 | 2 | *trnP-UGG*, *trnW-CCA* |
| - | 586779 | 590307 | 2 | *5SrRNA*, *26SrRNA* |
| - | 607004 | 609191 | 3 | *rps12*, *nad3*, *ccmC* |
| - | 633043 | 635244 | 3 | *5SrRNA*, *orf111-b*, *18SrRNA* |
| + | 638863 | 640251 | 5 | *trnS-GCU*, *trnF-GAA*, *orf106a*, *trnP-UGG*, *orf114* |
